# Publisher Correction: AI-NERD: Elucidation of relaxation dynamics beyond equilibrium through AI-informed X-ray photon correlation spectroscopy

**DOI:** 10.1038/s41467-024-52178-9

**Published:** 2024-09-09

**Authors:** James P. Horwath, Xiao-Min Lin, Hongrui He, Qingteng Zhang, Eric M. Dufresne, Miaoqi Chu, Subramanian K.R.S. Sankaranarayanan, Wei Chen, Suresh Narayanan, Mathew J. Cherukara

**Affiliations:** 1 Advanced Photon Source, Argonne National Laboratory, Lemont, IL USA; 2 Center for Nanoscale Materials, Argonne National Laboratory, Lemont, IL USA; 3 Materials Science Division and Center for Molecular Engineering, Argonne National Laboratory, Lemont, IL USA; 4 Pritzker School of Molecular Engineering, University of Chicago, Chicago, IL USA; 5 Department of Mechanical and Industrial Engineering, University of Illinois, Chicago, IL USA

**Keywords:** Characterization and analytical techniques, Scientific data

Correction to: *Nature Communications* 10.1038/s41467-024-49381-z, published online 15 July 2024

The original version of this Article incorrectly omitted James P. Horwath and Suresh Narayanan as corresponding authors. This has now been corrected in both the PDF and HTML versions of the Article.

