# Supplementary material for: AI-NERD: Elucidation of relaxation dynamics beyond equilibrium through AI-informed X-ray photon correlation spectroscopy
Source: Nat Commun. 2024 Jul 15;15:5945. doi: 10.1038/s41467-024-49381-z (PMC11251071; doi:10.1038/s41467-024-49381-z)
Supplement: Supplementary file 1 — Supplementary Information [file 41467_2024_49381_MOESM1_ESM.pdf]

Supplementary Information

Elucidation of Relaxation Dynamics Beyond Equilibrium Through AI-Informed X-ray Photon Correlation Spectroscopy

James P. Horwath[1], Xiao-Min Lin[2], Hongrui He[3,4], Qingteng Zhang[1], Eric M. Dufresne[1], Miaoqi Chu[1], Subramanian K.R.S Sankaranarayanan[2,5], Wei Chen[3,4], Suresh Narayanan[1], Mathew J. Cherukara[1]

1. Advanced Photon Source, Argonne National Laboratory, Lemont, IL 60439
2. Center for Nanoscale Materials, Argonne National Laboratory, Lemon, IL 60439
3. Materials Science Division and Center for Molecular Engineering, Argonne National Laboratory, Lemont, IL 60439
4. Pritzker School of Molecular Engineering, University of Chicago, Chicago, IL 60637
5. Department of Mechanical and Industrial Engineering, University of Illinois, Chicago, IL 60607

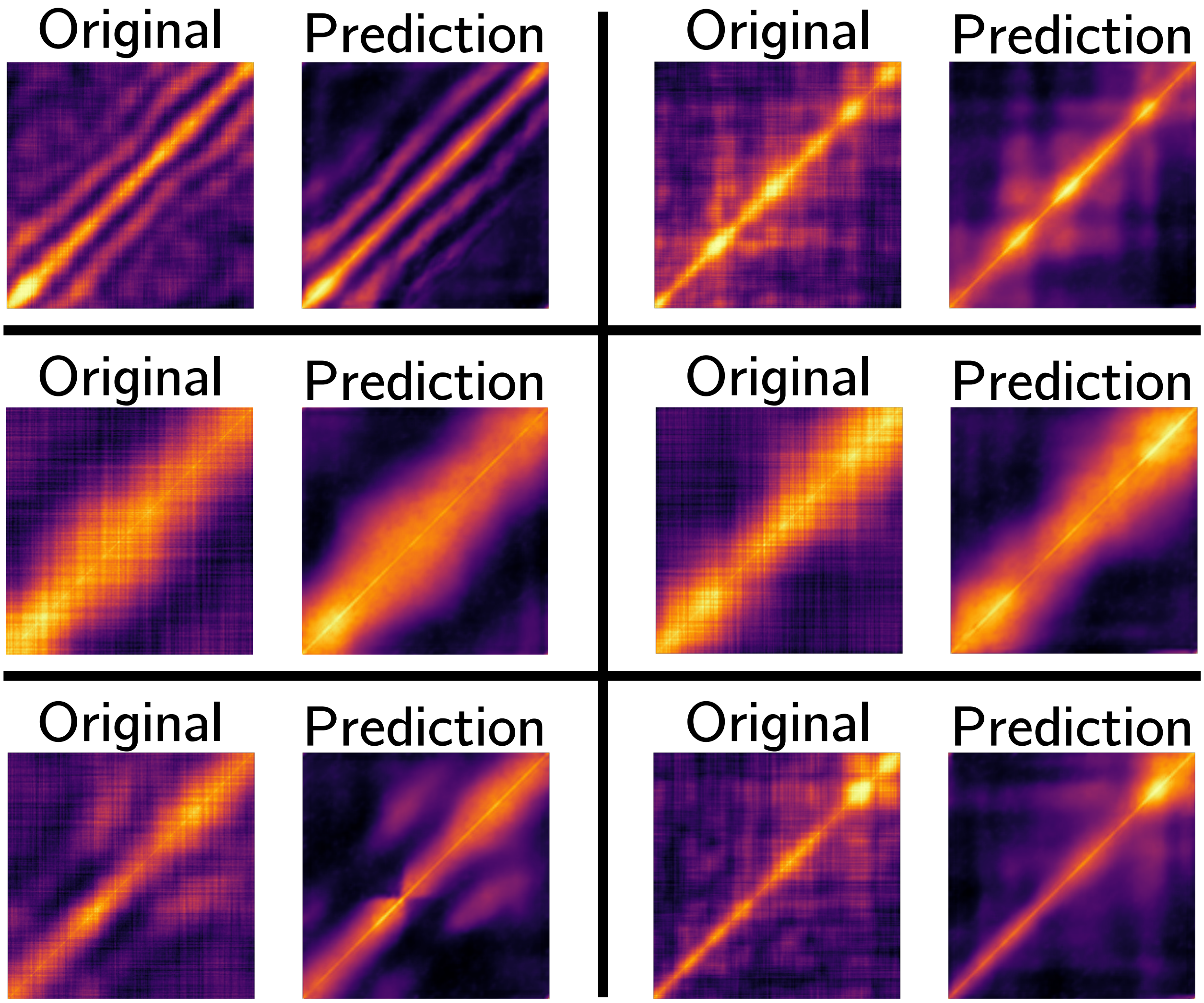

Supplemental Figure 1. Sample of experimental C2 and reproductions from the trained autoencoder. For each panel, the left image shows the raw data and the right image shows the neural network prediction.

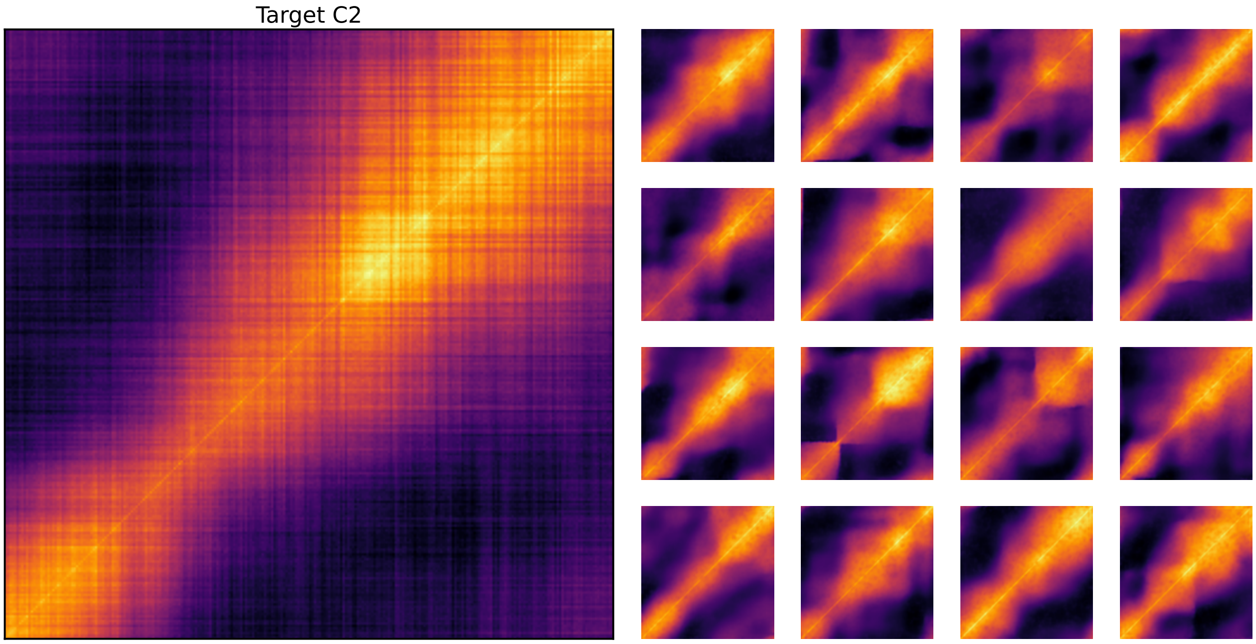

Supplemental Figure 2. Example of generative capabilities of the trained CNN. The latent representation of the target image was used as a baseline for generating new data. Random gaussian noise (sigma = 0.3) was added to the latent vector, and then decoded to yield the 16 unique samples at the left. While some artificial data appear more realistic than others, most capture the narrowing and subsequent expansion of the correlation width throughout the C2.

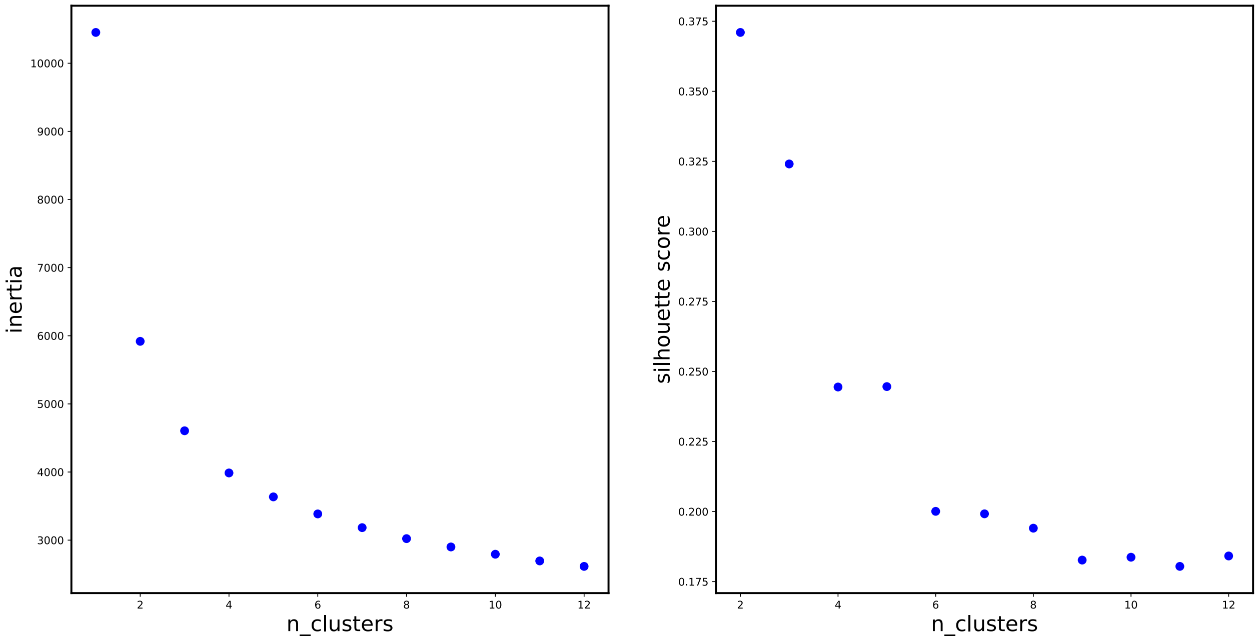

Supplemental Figure 3. Elbow plot tracking KMeans Clustering inertia and silhouette score as a function of number of clusters.

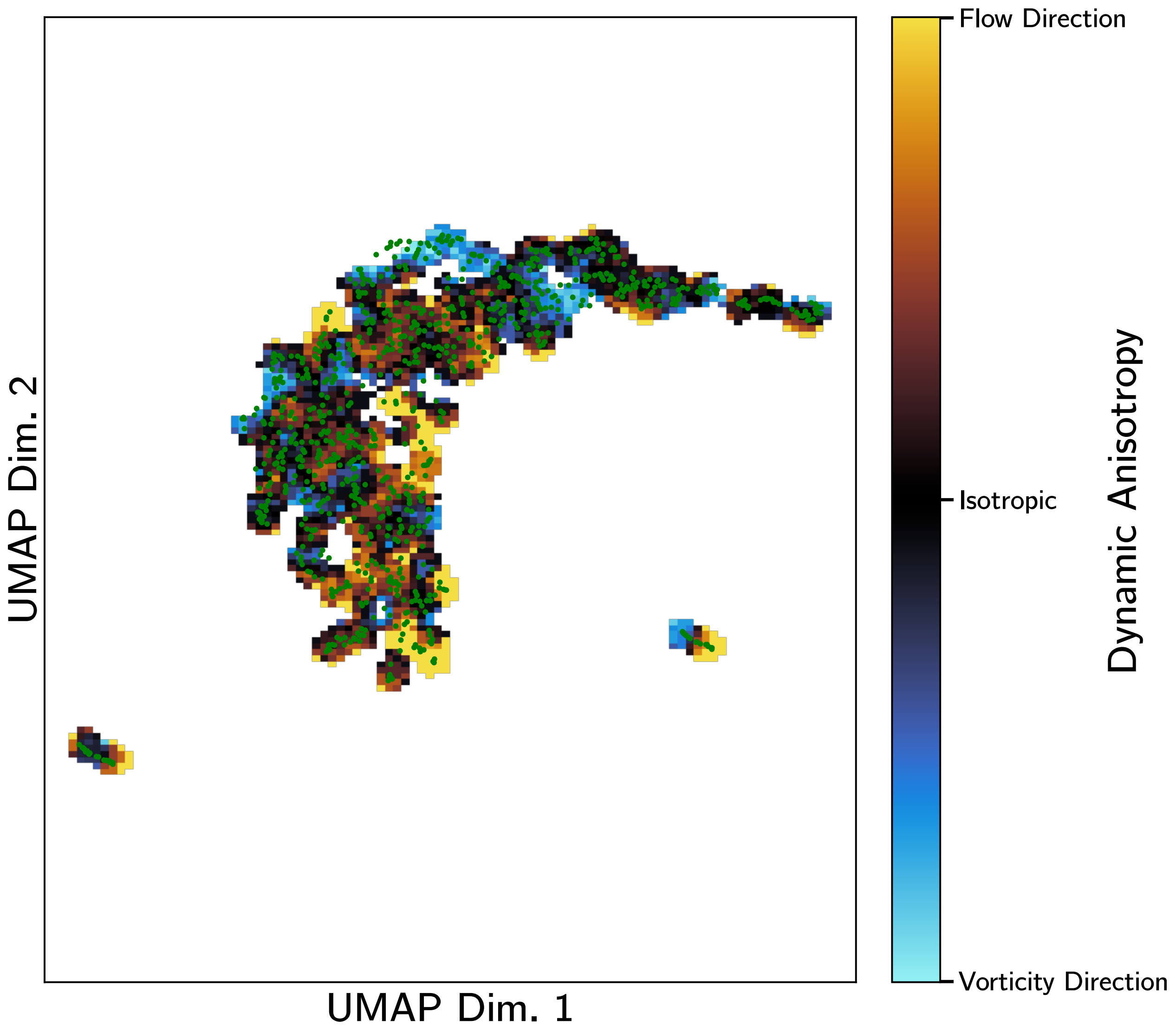

Supplemental Figure 4. Map showing the distribution of flow and vorticity direction C2 in the latent space. To determine this, the proportion of flow direction C2 to the total number of C2 within a specified radius is counted and termed 'Dynamic Anisotropy', i.e. the region of the latent space contains isotropic dynamics is the proportion of flow direction dynamics in the region is 0.5, and dynamics are dominated by the vorticity and flow directions if the proportions are 0 and 1, respectively. This map shows that vorticity direction dynamics are clustered in the top portion of the latent space, signifying that specific C2 patterns occur only in specific scattering directions.

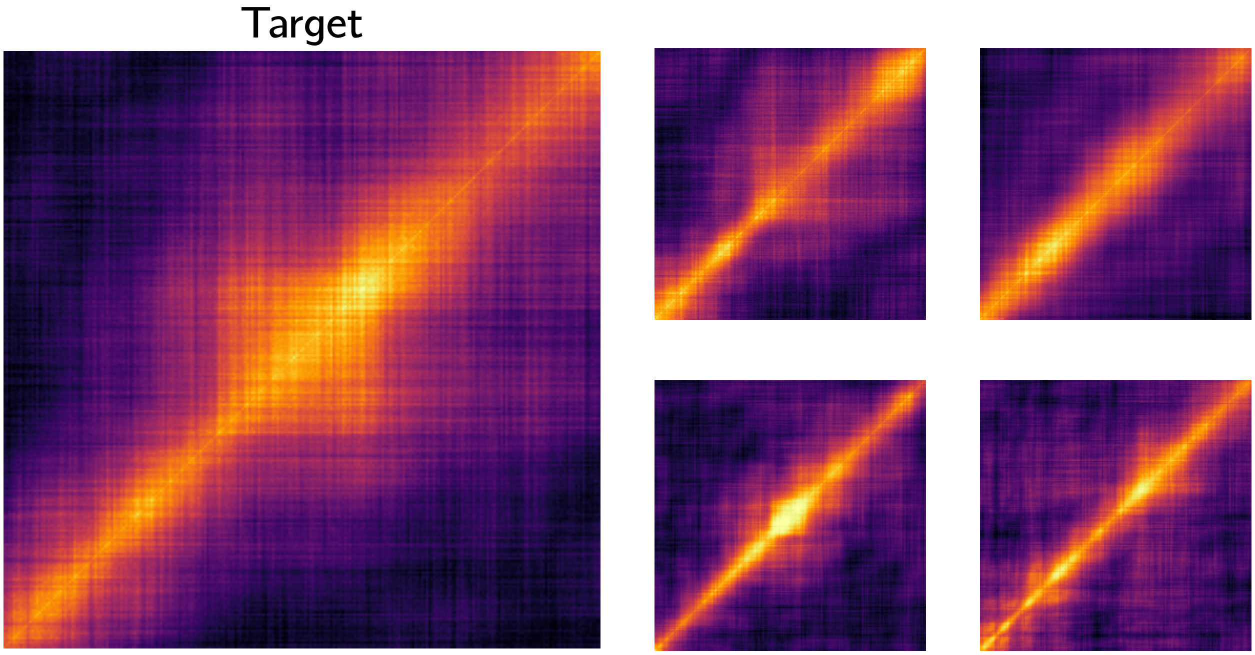

Supplemental Figure 5. Example of similarity analysis where nearest neighbors are less similar to the target than shown in Figure 4 of the main text.

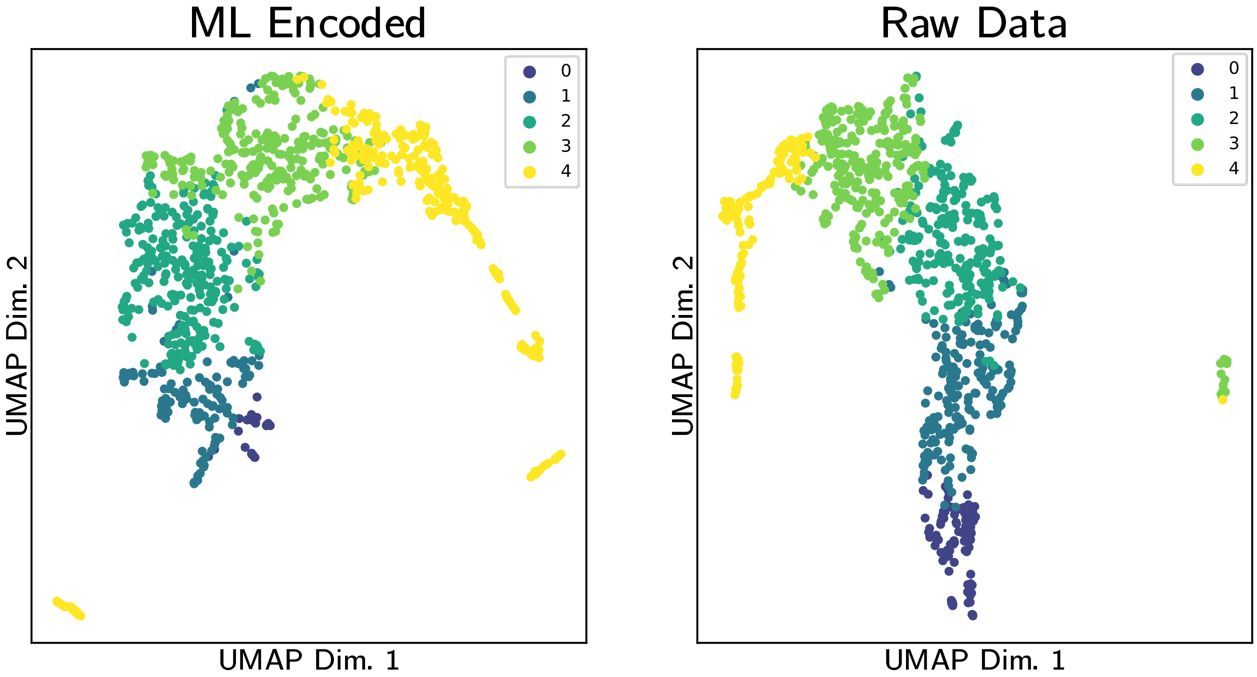

Supplemental Figure 6. Comparison of UMAP projection and clustering results between operating on raw C2 data and the ML latent space.

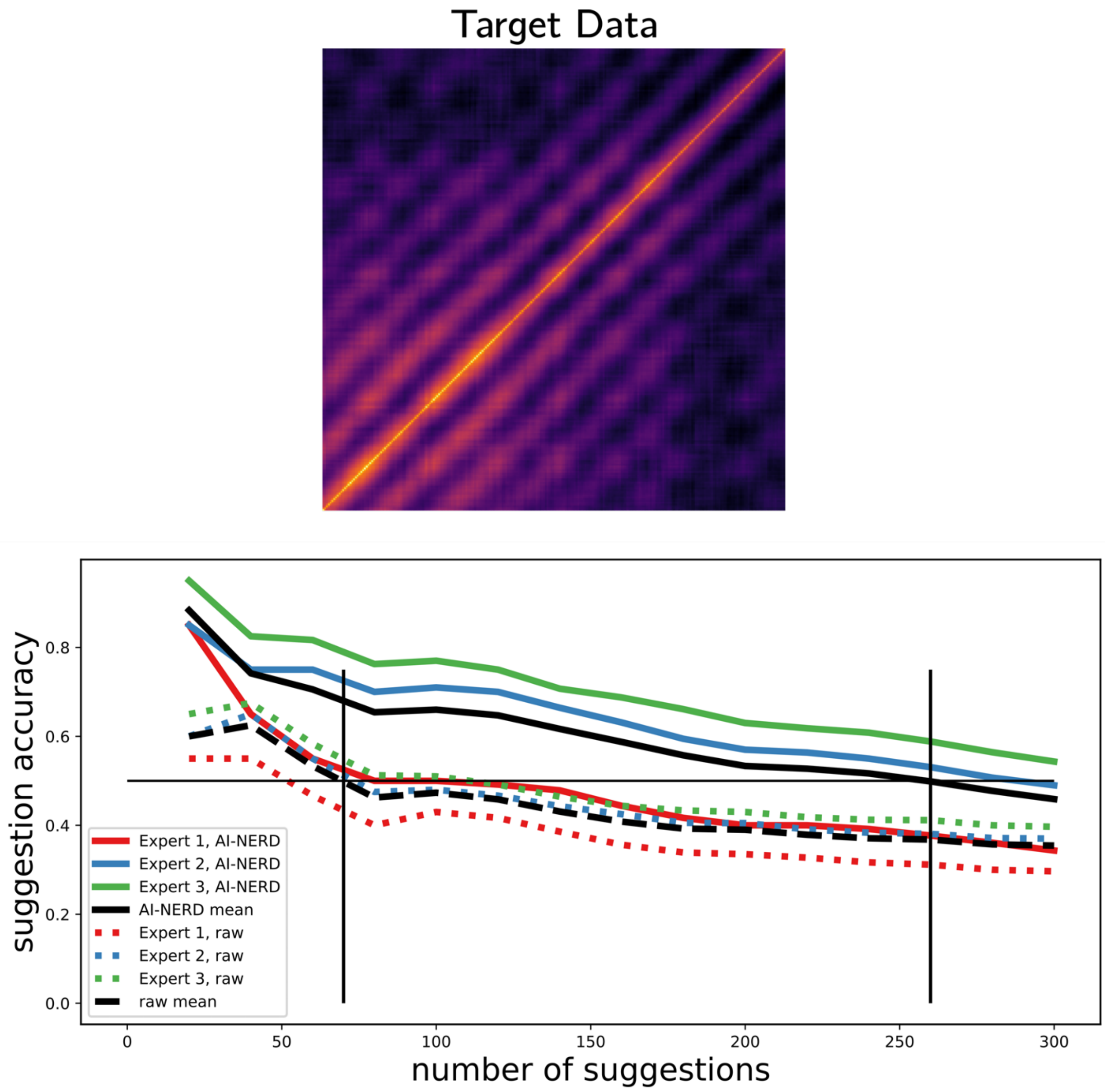

Supplemental Figure 7. A blind test to evaluate the accuracy of AI-NERD. Three XPCS experts were given a target C2 image and two unlabeled datasets consisting of 300 unique C2 – one set was generated using AI-NERD, and the other was generated from raw data based on the proximity to the target in the latent space (see Supplementary Figure 6). The experts were asked to go through each set and identify which samples showed similar features to the target. Suggestion accuracy is defined as the fraction of C2 which experts identified as similar to the target. Accuracy is plotted as a function of the number of C2 encountered in nearest-neighbor order to track how visual similarity changes with Euclidean distance in the AI-NERD and raw data spaces.

```
----------------------------------------------------------------
        Layer (type)               Output Shape         Param #
================================================================
            Conv2d-1        [-1, 16, 256, 256]             160
           Dropout-2        [-1, 16, 256, 256]               0
              ReLU-3        [-1, 16, 256, 256]               0
            Conv2d-4        [-1, 16, 256, 256]           2,320
           Dropout-5        [-1, 16, 256, 256]               0
              ReLU-6        [-1, 16, 256, 256]               0
         MaxPool2d-7          [-1, 16, 64, 64]               0
            Conv2d-8          [-1, 32, 64, 64]           4,640
           Dropout-9          [-1, 32, 64, 64]               0
             ReLU-10          [-1, 32, 64, 64]               0
           Conv2d-11          [-1, 32, 64, 64]           9,248
          Dropout-12          [-1, 32, 64, 64]               0
             ReLU-13          [-1, 32, 64, 64]               0
        MaxPool2d-14          [-1, 32, 16, 16]               0
           Conv2d-15          [-1, 64, 16, 16]          18,496
          Dropout-16          [-1, 64, 16, 16]               0
             ReLU-17          [-1, 64, 16, 16]               0
           Conv2d-18          [-1, 64, 16, 16]          36,928
          Dropout-19          [-1, 64, 16, 16]               0
             ReLU-20          [-1, 64, 16, 16]               0
        MaxPool2d-21            [-1, 64, 4, 4]               0
          Flatten-22                 [-1, 1024]               0
           Linear-23                   [-1, 64]          65,600
             ReLU-24                   [-1, 64]               0
           Linear-25                 [-1, 1024]          66,560
             ReLU-26                 [-1, 1024]               0
        Unflatten-27            [-1, 64, 4, 4]               0
           Conv2d-28            [-1, 64, 4, 4]          36,928
          Dropout-29            [-1, 64, 4, 4]               0
             ReLU-30            [-1, 64, 4, 4]               0
           Conv2d-31            [-1, 64, 4, 4]          36,928
          Dropout-32            [-1, 64, 4, 4]               0
             ReLU-33            [-1, 64, 4, 4]               0
         Upsample-34          [-1, 64, 16, 16]               0
           Conv2d-35          [-1, 64, 16, 16]          36,928
          Dropout-36          [-1, 64, 16, 16]               0
             ReLU-37          [-1, 64, 16, 16]               0
           Conv2d-38          [-1, 64, 16, 16]          36,928
          Dropout-39          [-1, 64, 16, 16]               0
             ReLU-40          [-1, 64, 16, 16]               0
         Upsample-41          [-1, 64, 64, 64]               0
           Conv2d-42          [-1, 32, 64, 64]          18,464
          Dropout-43          [-1, 32, 64, 64]               0
             ReLU-44          [-1, 32, 64, 64]               0
           Conv2d-45          [-1, 32, 64, 64]           9,248
          Dropout-46          [-1, 32, 64, 64]               0
             ReLU-47          [-1, 32, 64, 64]               0
         Upsample-48        [-1, 32, 256, 256]               0
           Conv2d-49         [-1, 1, 256, 256]             289
          Sigmoid-50         [-1, 1, 256, 256]               0
================================================================
Total params: 379,665
Trainable params: 379,665
Non-trainable params: 0
----------------------------------------------------------------
Input size (MB): 0.25
Forward/backward pass size (MB): 81.27
Params size (MB): 1.45
Estimated Total Size (MB): 82.97
----------------------------------------------------------------
```

Supplemental Figure 8. Explicit description of the CNN Architecture used for this research generated using the torch-summary python package.

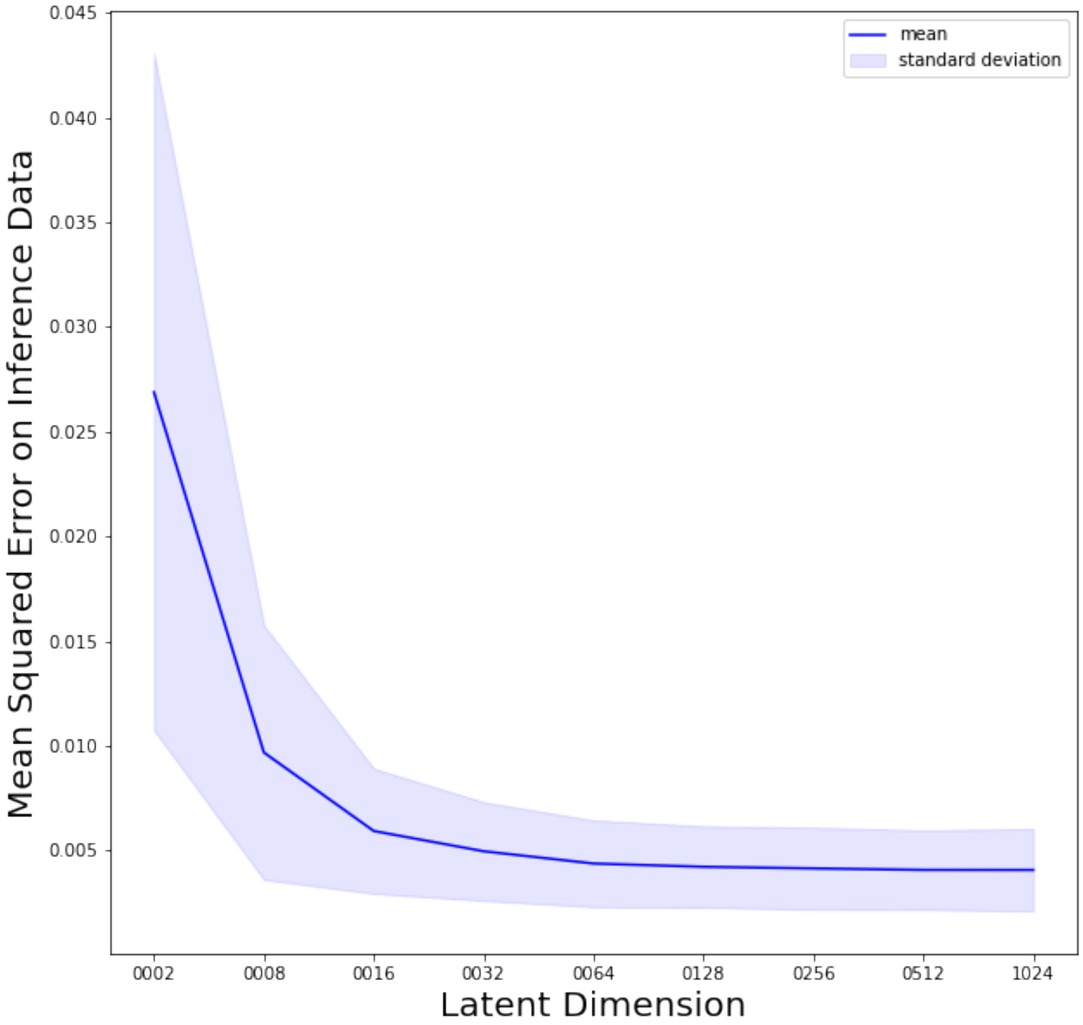

Supplemental Figure 9. Plot showing mean squared error for C2 reconstruction on the test dataset as a function of latent dimension.
