# Supplementary material for: AI-NERD: Elucidation of relaxation dynamics beyond equilibrium through AI-informed X-ray photon correlation spectroscopy
Source: Nat Commun. 2024 Jul 15;15:5945. doi: 10.1038/s41467-024-49381-z (PMC11251071; doi:10.1038/s41467-024-49381-z)
Supplement: Supplementary file 3 — Reporting Summary [file 41467_2024_49381_MOESM3_ESM.pdf]

nature portfolio

Corresponding author(s): Mathew J. Cherukara

Last updated by author(s): Apr 23, 2024

Reporting Summary

Nature Portfolio wishes to improve the reproducibility of the work that we publish. This form provides structure for consistency and transparency in reporting. For further information on Nature Portfolio policies, see our Editorial Policies and the Editorial Policy Checklist.

Statistics

For all statistical analyses, confirm that the following items are present in the figure legend, table legend, main text, or Methods section.

n/a	Confirmed	
x	☐	The exact sample size (*n*) for each experimental group/condition, given as a discrete number and unit of measurement
x	☐	A statement on whether measurements were taken from distinct samples or whether the same sample was measured repeatedly
x	☐	The statistical test(s) used AND whether they are one- or two-sided<br>*Only common tests should be described solely by name; describe more complex techniques in the Methods section.*
x	☐	A description of all covariates tested
x	☐	A description of any assumptions or corrections, such as tests of normality and adjustment for multiple comparisons
x	☐	A full description of the statistical parameters including central tendency (e.g. means) or other basic estimates (e.g. regression coefficient) AND variation (e.g. standard deviation) or associated estimates of uncertainty (e.g. confidence intervals)
x	☐	For null hypothesis testing, the test statistic (e.g. *F*, *t*, *r*) with confidence intervals, effect sizes, degrees of freedom and *P* value noted<br>*Give P values as exact values whenever suitable.*
x	☐	For Bayesian analysis, information on the choice of priors and Markov chain Monte Carlo settings
x	☐	For hierarchical and complex designs, identification of the appropriate level for tests and full reporting of outcomes
x	☐	Estimates of effect sizes (e.g. Cohen's *d*, Pearson's *r*), indicating how they were calculated

Our web collection on statistics for biologists contains articles on many of the points above.

Software and code

Policy information about availability of computer code

Data collection	Data were collected at the Advanced Photon Source, beamline 8-ID-I using custom built x-ray scattering acquisition software.
Data analysis	All analysis was carried out using open source python packages. Code needed to exactly reproduce this analysis has been made available with an open source license at https://github.com/jhorwath/XPCS_Clustering.

For manuscripts utilizing custom algorithms or software that are central to the research but not yet described in published literature, software must be made available to editors and reviewers. We strongly encourage code deposition in a community repository (e.g. GitHub). See the Nature Portfolio guidelines for submitting code & software for further information.

Data

Policy information about availability of data

All manuscripts must include a data availability statement. This statement should provide the following information, where applicable:

- Accession codes, unique identifiers, or web links for publicly available datasets
- A description of any restrictions on data availability
- For clinical datasets or third party data, please ensure that the statement adheres to our policy

Raw XPCS and rheology data is available at https://anl.box.com/s/dhqahh467gnv0srz0tct1ymaofgr07te.

Research involving human participants, their data, or biological material

Policy information about studies with human participants or human data. See also policy information about sex, gender (identity/presentation), and sexual orientation and race, ethnicity and racism.

Reporting on sex and gender	n/a
Reporting on race, ethnicity, or other socially relevant groupings	n/a
Population characteristics	n/a
Recruitment	n/a
Ethics oversight	n/a

Note that full information on the approval of the study protocol must also be provided in the manuscript.

Field-specific reporting

Please select the one below that is the best fit for your research. If you are not sure, read the appropriate sections before making your selection.

☐ Life sciences ☐ Behavioural & social sciences ☒ Ecological, evolutionary & environmental sciences

For a reference copy of the document with all sections, see nature.com/documents/nr-reporting-summary-flat.pdf

Ecological, evolutionary & environmental sciences study design

All studies must disclose on these points even when the disclosure is negative.

Study description	Rheo-XPCS data were collected to track relaxation dynamics in a silica-polyethylene gycol suspension. Machine learning analysis was used to automate the process of extracting physical information from complex XPCS two-time correlation functions.
Research sample	Material samples consisted of suspensions of silica spheres in polyethylene glycol solution with varying solid volume fractions.
Sampling strategy	Sample sizes were not calculated for this research, as we deal with information in the big-data limit. Our research processes over 10,000 images to identify qualitative trends and prototypical behavior in a dynamic sample.
Data collection	X-ray scattering data collection and XPCS correlation calculations were performed at the Advanced Photon Source by HongRui He, Xiao-Min Lin, and Suresh Narayanan.
Timing and spatial scale	Data were collected over the course of December 8-12, 2021. Data were captured in several separate x-ray scattering experiments to induce different shear histories. Standard experiments collected data over the course of an hour, collecting x-ray scattering frames at 100 frames per second.
Data exclusions	No data were excluded
Reproducibility	All machine learning analyses are reproducible based on the raw data and analysis codes provided.
Randomization	Randomization was not relevant to this research. All data that was collected was processed, and the time-resolved nature of the data required that it stayed in specific order.
Blinding	Blinding was only relevant for the results of Supplemental Figure 7, where scientific experts evalutated the accuracy of machine learning predictions. This was conducted in a single blind fashion - experts were unaware of how predictions were generated.

Did the study involve field work? ☐ Yes ☒ No

Reporting for specific materials, systems and methods

We require information from authors about some types of materials, experimental systems and methods used in many studies. Here, indicate whether each material, system or method listed is relevant to your study. If you are not sure if a list item applies to your research, read the appropriate section before selecting a response.

Materials & experimental systems

n/a	Involved in the study
☒	☐ Antibodies
☒	☐ Eukaryotic cell lines
☒	☐ Palaeontology and archaeology
☒	☐ Animals and other organisms
☒	☐ Clinical data
☒	☐ Dual use research of concern
☒	☐ Plants

Methods

n/a	Involved in the study
☒	☐ ChIP-seq
☒	☐ Flow cytometry
☒	☐ MRI-based neuroimaging

Plants

Seed stocks	n/a
Novel plant genotypes	n/a
Authentication	n/a
